# Involvement of two putative gene clusters for exopolysaccharide biosynthesis and modification in sporangium formation in *Actinoplanes missouriensis*

**DOI:** 10.1128/aem.02330-25

**Published:** 2026-06-11

**Authors:** Takeaki Tezuka, Yasuo Ohnishi

**Affiliations:** 1 Department of Biotechnology, Graduate School of Agricultural and Life Sciences, The University of Tokyo 13143, Bunkyo-ku, Tokyo, Japan; 2 Collaborative Research Institute for Innovative Microbiology, The University of Tokyo, Bunkyo-ku, Tokyo, Japan; Washington University in St Louis, St. Louis, Missouri, USA

**Keywords:** *Actinoplanes missouriensis*, exopolysaccharide, GtrA, sporangium formation, Wzx/Wzy-dependent pathway

## Abstract

The terminal sporangium of the actinomycete *Actinoplanes missouriensis* contains a few hundred spores surrounded by a matrix substance, which is mainly composed of a polysaccharide synthesized by the *imp* gene cluster. However, the involvement of other extracellular polysaccharide (EPS) biosynthetic gene clusters in sporangium matrix formation remains to be examined. In this study, we characterized four putative EPS biosynthetic gene clusters by gene disruption, which were transcribed during sporangium formation and were thereby expected to be involved in the production or modification of EPSs inside sporangia. An *in silico* analysis proposed that three gene clusters, *wps*-1 (*AMIS_7280–AMIS_7380*), *wps*-2 (*AMIS_56690–AMIS_56780*), and *wps*-3 (*AMIS_58080–AMIS_58180*), provide the Wzx/Wzy-dependent pathway, and that another gene cluster, *gsf* (*AMIS_70380–AMIS_70480*), provides the GtrA-dependent pathway. Severely distorted sporangia were observed by scanning electron microscopy in the null mutant of the whole *wps*-1 cluster (Δ*wps*-1 strain). The null mutant of the whole *gsf* cluster (Δ*gsf* strain) formed sporangia smaller than the wild-type sporangia. Transmission electron microscopy revealed that the spores inside the Δ*gsf* sporangia were not fully matured. Spore release did not occur in the Δ*wps*-1 strain under appropriate aquatic conditions, whereas sporangia of the Δ*gsf* strain opened to release spores under the same conditions. Disruption of each gene in the *wps*-1 cluster indicated that all genes, except *wpsD* (*AMIS_7310*), were indispensable for normal sporangium formation. We concluded that exopolysaccharide production and modification mediated by the *wps*-1 and *gsf* clusters, as well as the *imp* cluster, play crucial roles in sporangium formation in *A. missouriensis*.

**IMPORTANCE:**

*Actinoplanes missouriensis* forms terminal sporangia, which consist of a sporangium envelope, spores, and a matrix substance. The sporangium matrix encapsulates the spores inside the sporangium. Recently, we reported that a major component of the sporangium matrix is a polysaccharide consisting of repeating oligosaccharides and that a gene cluster, named *imp*, is involved in the production of the polysaccharide. In the present study, through a gene disruption experiment, we revealed that two gene clusters, named *wps*-1 and *gsf*, are also required for normal sporangium formation in *A. missouriensis*, providing important insights into the components of the sporangium matrix. The *wps*-1 cluster appears to be responsible for producing sulfated polysaccharide(s). Sulfated polysaccharides are produced by animals and algae but are not common in bacteria, except cyanobacteria. Thus, although experimental verification is required, this study raises the possibility that the ability to produce sulfated polysaccharides is also present in phylogenetically distant actinomycetes.

## INTRODUCTION

Bacteria produce a wide variety of polysaccharides that contribute to various cellular functions. Intracellular polysaccharides provide cells with storage substances, such as glycogen (1). Capsular polysaccharides (CPSs) and lipopolysaccharides (LPSs) are closely associated with the cell surface and are important virulence factors in pathogenic bacteria (2). Extracellular polysaccharides (EPSs), which are secreted or synthesized in the surrounding environment, play important roles in biofilm formation, motility, and pathogenicity (3). Bacterial EPSs are produced by four general mechanisms: (i) the Wzx/Wzy-dependent pathway, (ii) ATP-binding cassette (ABC) transporter-dependent pathway, (iii) synthase-dependent pathway, and (iv) extracellular synthesis catalyzed by sucrase proteins. In the Wzx/Wzy-dependent pathway, oligosaccharide repeating units are assembled on the lipid carrier undecaprenyl pyrophosphate by glycosyltransferases inside the cell and exported from the inner leaflet to the outer leaflet of the plasma membrane by a flippase (Wzx protein). Subsequently, polymerization of the repeating units occurs on the outer leaflet of the membrane by a polymerase (Wzy protein). The length of the produced polysaccharide strand is controlled by a polysaccharide co-polymerase (Wzz protein) (4).

In gram-positive bacteria, a multi-component transmembrane glycosylation system is involved in the extracellular glycosylation of lipoteichoic acids (LTAs), wall teichoic acids (WTAs), and secondary cell wall polysaccharides (SCWPs) (5). The prototype of this system comprises a membrane-linked GT-A-fold glycosyltransferase, GtrA-type flippase, and membrane-spanning GT-C-fold glycosyltransferase (6). GT-A-fold glycosyltransferases produce a lipid-linked sugar intermediate, which is moved from the cytosolic layer to the extracellular layer by a GtrA-type flippase (7). GtrA-type flippases are small membrane proteins with 3–4 transmembrane helices and likely function as homodimers (8). GT-C-fold glycosyltransferases transfer the sugar from the lipid-linked intermediate to cell wall polymers (6, 7, 9). Similar extracellular glycosylation systems are involved in the periplasmic modification of LPSs and O-antigens in gram-negative bacteria (5).

Animals and algae produce EPSs containing sulfate groups, such as heparan sulfate and carrageenan (10). Insofar as bacteria, sulfated polysaccharides have been reported exclusively in cyanobacteria. Examples of cyanobacterial sulfated polysaccharides include synechan from *Synechocystis* sp. PCC 6803 and spirulan from *Arthrospira platensis* (11, 12). Sulfated polysaccharides are industrially important due to their potential applications in food, medicine, and other biomaterials (13, 14).

*Actinoplanes missouriensis* is a gram-positive, soil-inhabiting bacterium and a model actinomycete of the genus *Actinoplanes*. Under laboratory conditions, it forms branched substrate mycelia during vegetative growth, followed by the formation of globose and subglobose terminal sporangia from substrate mycelia through short sporangiophores. Each sporangium contains a few hundred spherical spores. The space among the spores inside a sporangium is filled with an intrasporangial matrix (named sporangium matrix), and the sporangium envelope is composed of three layers (15, 16). In response to water exposure, the sporangium envelope breaks to release spores through a process referred to as sporangium dehiscence (17, 18). After release from sporangia, spores swim as zoospores using flagella (19, 20). Zoospores exhibit chemotactic properties and stop swimming to germinate in a niche that is suitable for vegetative growth (21–23). On humic acid-trace element (HAT) agar, mature sporangia that can release spores under sporangium dehiscence-inducing conditions are formed after 5–7 days of cultivation at 30°C (24).

**Fig 1:**
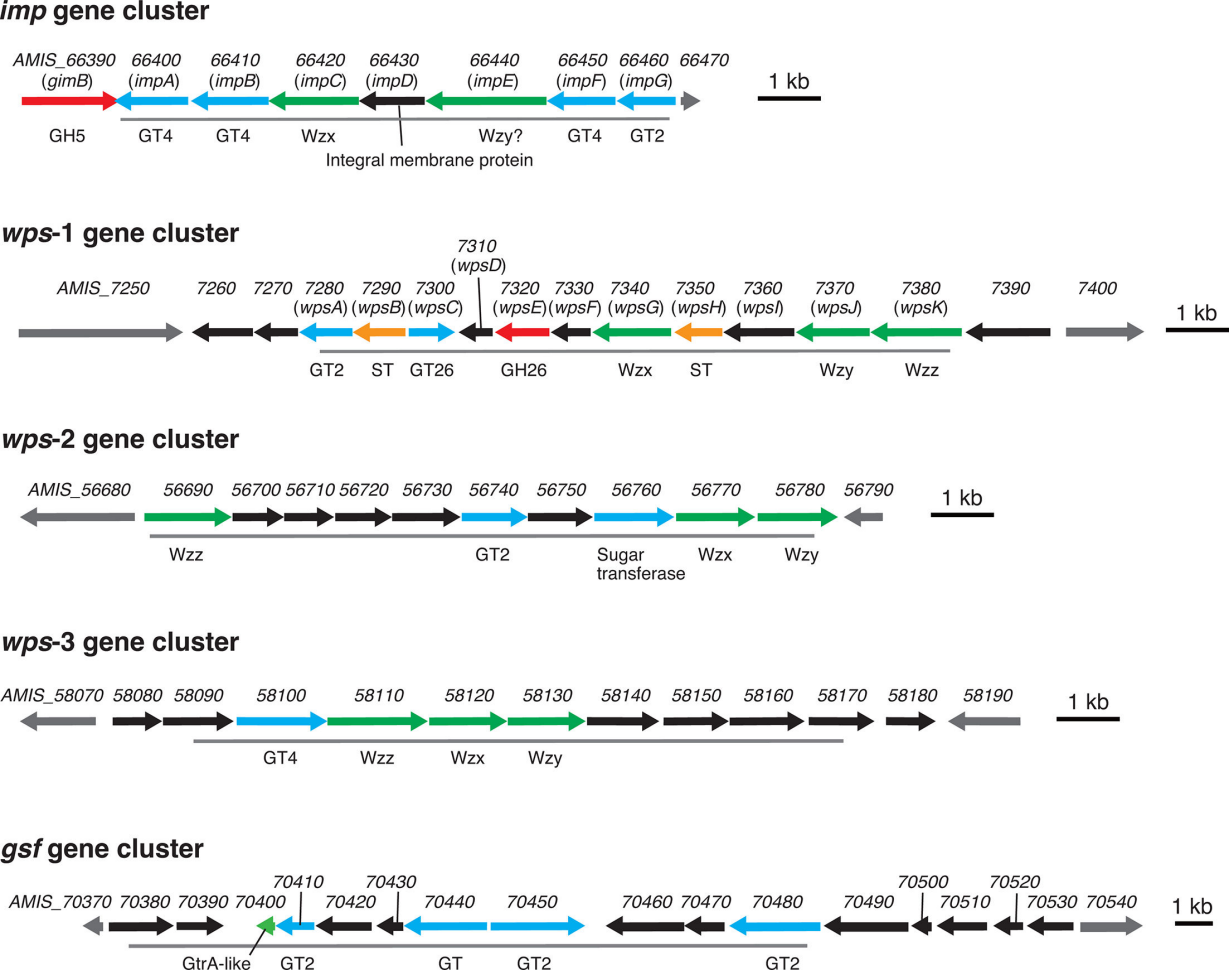
Gene organization of the *imp*, *wps*-1, *wps*-2, *wps*-3, and *gsf* clusters. Arrows indicate the locations of the open reading frames, including their lengths and directions. Genes encoding glycosyltransferases (or sugar transferases) are colored light blue. Genes encoding the Wzx/Wzy-dependent and GtrA-dependent pathways are shown in green. Sulfotransferase and glycosyl hydrolase genes are colored orange and red, respectively. Gene identification numbers are shown above the arrows. The gene names are shown in parentheses. The deleted region in each cluster deletion mutant is shown by a gray line below the arrows.

Recently, we reported that two glycoside hydrolases, GimA and GimB, decompose the sporangium matrix to release spores during sporangium dehiscence (18). In the same study, we found that a gene cluster (named *imp* cluster; *AMIS_66400–AMIS_66460*, Fig. 1) was responsible for sporangium matrix biosynthesis. The gene products of the *imp* cluster were expected to provide a Wzx/Wzy-dependent apparatus. Furthermore, hydrolysis of the sporangium matrix by GimA or GimB yielded an oligosaccharide containing four to eight sugars. Interestingly, an oligosaccharide of approximately half the length (between two and four sugars) was also produced by the hydrolysis of the sporangium matrix with both GimA and GimB, indicating that GimA and GimB cleave the polysaccharide at different positions in the oligosaccharide unit. Thus, we concluded that a major component of the sporangium matrix is a polysaccharide consisting of repeating oligosaccharides, although its chemical structure remains unknown (18). In the present study, we characterized four gene clusters that were expected to be involved in EPS production and modification to obtain further insights into the components of *A. missouriensis* sporangia. Three gene clusters provide the Wzx/Wzy-dependent pathway, whereas the remaining one provides the GtrA-dependent pathway. Through gene disruption experiments, we demonstrated that two of the four gene clusters, in addition to the *imp* gene cluster, play crucial roles in sporangium formation.

## RESULTS

### *In silico* analysis of the four gene clusters

We focused on the following gene clusters, which are transcribed during sporangium formation (see below), on the *A. missouriensi*s chromosome: (i) *wps* (Wzx/Wzy-dependent polysaccharide biosynthesis) gene cluster 1 (*wps*-1) comprising 11 genes (*AMIS_7280–AMIS_7380*; Fig. 1, Fig. S1A), (ii) *wps* gene cluster 2 (*wps*-2) comprising 10 genes (*AMIS_56690–AMIS_56780*; Fig. 1, Fig. S2A), (iii) *wps* gene cluster 3 (*wps*-3) comprising 11 genes (*AMIS_58080–AMIS_58180*; Fig. 1, Fig. S3A), and (iv) the *gsf* (GtrA-dependent pathway involved in sporangium formation) gene cluster comprising 11 genes (*AMIS_70380–AMIS_70480*; Fig. 1, Fig. S4A).

#### *wps*-1 gene cluster

We named AMIS_7280–AMIS_7380 WpsA–WpsK (Fig. S1B). A protein database search using InterPro ver. 107.0 (https://www.ebi.ac.uk/interpro/) predicted that WpsA harbors a glycosyltransferase family 2 domain (accession number IPR050834; residues 3–282), and that WpsC harbors a WecG/TagA/CpsF family glycosyltransferase domain (IPR004629; residues 64–241) (Fig. S1B). WpsE was predicted to harbor a glycoside hydrolase family 26 domain (IPR022790; residues 38–320, Fig. S1B). According to the CAZy database (https://www.cazy.org), WpsA, WpsC, and WpsE belong to GT2, GT26, and GH26 enzyme families, respectively. WpsG was predicted to harbor a polysaccharide biosynthesis and transport domain (IPR050833; residues 2–471, Fig. S1B). WpsJ was predicted to harbor an O-antigen ligase-like domain (IPR051533; residues 37–442, Fig. S1B). The N- and C-terminal portions of WpsK were predicted to harbor a polysaccharide chain length determinant N-terminal domain (IPR003856; residues 10–68) and a bacterial polysaccharide biosynthesis and export domain (IPR050445; residues 164–496), respectively (Fig. S1B). According to the domain organization, we expected that WpsG, WpsJ, and WpsK function as Wzx, Wzy, and Wzz proteins, respectively. It should be noted that two sulfotransferases in different families appeared to be encoded in this gene cluster (Fig. S1B): WpsB was predicted to harbor a Gal/GlcNAc/GalNAc sulfotransferase domain (IPR051135; residues 126–304), whereas WpsH was predicted to harbor a heparan sulfate sulfotransferase domain (IPR037359; residues 28–290). WpsF was predicted to harbor a SAM-dependent methyltransferase domain (IPR029063; residues 6–203, Fig. S1B). WpsI was predicted to harbor a protein kinase-like domain (IPR011009; residues 175–329, Fig. S1B). WpsD was predicted to harbor a domain of unknown function (DUF4262) (IPR025358; residues 27–152, Fig. S1B). The TMHMM 2.0 server (https://services.healthtech.dtu.dk/services/TMHMM-2.0/) predicted that WpsG, WpsJ, and WpsK contain 13, 10, and 1 transmembrane helices, respectively (Fig. S1B). Overall, the gene products of the *wps*-1 gene cluster were predicted to produce a sulfated exopolysaccharide(s) through the Wzx/Wzy-dependent pathway.

#### *wps*-2 gene cluster

AMIS_56740 was predicted to harbor a glycosyltransferase 2-like domain (IPR001173; residues 7–133), and the C-terminal portion of AMIS_56760 was predicted to harbor a bacterial sugar transferase domain (IPR003362; residues 220–412) (Fig. S2B). According to the CAZy database, AMIS_56740 belongs to the GT2 enzyme family, whereas AMIS_56760 is not listed as a glycosyltransferase. The N-terminal portion of AMIS_56760 was predicted to harbor a Gcn5-related *N*-acetyltransferase (GNAT) domain (IPR000182; residues 116–181, Fig. S2B). AMIS_56770 was predicted to harbor a polysaccharide biosynthesis and transport domain (IPR050833; residues 11–409, Fig. S2B). AMIS_56780 was predicted to harbor an O-antigen ligase-like domain (IPR051533; residues 16–391, Fig. S2B). The N- and C-terminal portions of AMIS_56690 were predicted to harbor a polysaccharide chain length determinant N-terminal domain (IPR003856; residues 3–82) and a bacterial polysaccharide biosynthesis and export domain (IPR050445; residues 145–429), respectively (Fig. S2B). Therefore, we expected that AMIS_56770, AMIS_56780, and AMIS_56690 function as the Wzx, Wzy, and Wzz proteins, respectively. AMIS_56700 was predicted to harbor a SAM-dependent methyltransferase domain (IPR029063; residues 12–251, Fig. S2B). AMIS_56710 was predicted to harbor a transketolase N-terminal domain (IPR005474; residues 8–263, Fig. S2B). AMIS_56720 was predicted to harbor transketolase pyrimidine-binding and C-terminal domains (IPR005475 and IPR033248; residues 11–158 and 183–297, respectively, Fig. S2B). AMIS_56730 and AMIS_56750 were predicted to harbor a DegT/DnrJ/EryC1/StrS family aminotransferase domain (IPR000653; residues 28–367 and 10–335, respectively, Fig. S2B). The TMHMM 2.0 server predicted that AMIS_56690, AMIS_56740, AMIS_56770, and AMIS_56780 contain 1, 3, 11, and 10 transmembrane helices, respectively (Fig. S2B). Overall, the gene products of the *wps*-2 gene cluster were predicted to produce an EPS(s) through the Wzx/Wzy-dependent pathway.

#### *wps*-3 gene cluster

The N- and C-terminal portions of AMIS_58100 were predicted to harbor a glycosyltransferase subfamily 4-like N-terminal domain (IPR028098; residues 20–190) and glycosyltransferase family 1 domain (IPR001296; residues 207–376), respectively (Fig. S3B). According to the CAZy database, this protein belongs to the GT4 enzyme family. AMIS_58120 was predicted to harbor a polysaccharide biosynthesis and transport domain (IPR050833; residues 25–409, Fig. S3B). AMIS_58130 was predicted to harbor an O-antigen ligase-like domain (IPR051533; residues 37–385, Fig. S3B). AMIS_58110 was predicted to harbor a bacterial polysaccharide biosynthesis and export domain (IPR050445; residues 146–420, Fig. S3B). Therefore, we expected that AMIS_58120, AMIS_58130, and AMIS_58110 function as Wzx, Wzy, and Wzz proteins, respectively. AMIS_58080 was predicted to harbor a polysaccharide deacetylase domain (IPR051398; residues 50–242, Fig. S3B). AMIS_58090 was predicted to harbor a DegT/DnrJ/EryC1/StrS family aminotransferase domain (IPR000653; residues 7–364, Fig. S3B). AMIS_58150 was predicted to harbor a BioF2-like acetyltransferase domain (IPR038740; residues 153–283, Fig. S3B). AMIS_58160 was predicted to harbor a PheA/TfdB family FAD-binding monooxygenase domain (IPR050631; residues 3–373, Fig. S3B). AMIS_58170 was predicted to harbor an acyl-CoA *N*-acyltransferase domain (IPR016181; residues 148-322, Fig. S3B). AMIS_58180 was predicted to harbor an intradiol ring-cleavage dioxygenase domain (IPR015889; residues 5–243, Fig. S3B). No functional domains were predicted in AMIS_58140. The TMHMM 2.0 server predicted that AMIS_58110, AMIS_58120, and AMIS_58130 contain 1, 12, and 11 transmembrane helices, respectively (Fig. S3B). Overall, the gene products of the *wps*-3 gene cluster were predicted to produce an EPS(s) through the Wzx/Wzy-dependent pathway.

#### *gsf* gene cluster

AMIS_70410 was predicted to harbor a glycosyltransferase 2 domain (IPR050256; residues 3–307, Fig. S4B). The C-terminal portion of AMIS_70440 was predicted to harbor a glycosyltransferase family 28 C-terminal domain (IPR007235; residues 619–685, Fig. S4B). AMIS_70450 and AMIS_70480 were predicted to harbor a glycosyltransferase 2-like domain (IPR001173; residues 205–317 and 214–324, respectively, Fig. S4B). According to the CAZy database, AMIS_70410, AMIS_70450, and AMIS_70480 belong to the GT2 enzyme family. AMIS_70440 is listed as a glycosyltransferase, but is not assigned to any enzyme family. AMIS_70400 was predicted to harbor a bacterial cell wall glycosylation GtrA domain (IPR051401; residues 5–121, Fig. S4B). AMIS_70380 was predicted to harbor a stealth family protein domain (IPR047141; residues 132–386, Fig. S4B). AMIS_70390 was predicted to harbor a UDP-*N*-acetyl-D-mannosamine/glucosamine dehydrogenase domain (IPR028359; residues 4–417, Fig. S4B). AMIS_70460 was predicted to harbor an acyltransferase 3 domain (IPR050879; residues 21–542, Fig. S4B). AMIS_70470 was predicted to harbor a SAM-dependent methyltransferase domain (IPR029063; residues 160–332, Fig. S4B). No functional domains were predicted for AMIS_70420 or AMIS_70430. The TMHMM 2.0 server predicted that AMIS_70400, AMIS_70410, AMIS_70420, AMIS_70460, and AMIS_70470 contain 4, 2, 11, 11, and 2 transmembrane helices, respectively (Fig. S4B). Overall, the gene products of the *gsf* gene cluster were predicted to provide a GtrA-dependent glycosylation apparatus.

### Transcriptional analysis of the four gene clusters

The transcription profiles of the four gene clusters were analyzed based on our previous RNA-Seq analysis (17). In *A. missouriensis*, the global transcriptional regulator TcrA controls sporangium formation, spore dormancy, and sporangium dehiscence by activating transcription of its regulon (25). In our previous study, the transcriptomes of wild-type and *tcrA* null mutant (Δ*tcrA*) strains were analyzed using RNA samples extracted from cells cultivated on HAT agar at 30°C for 6 days (25). Based on this result, we also examined the dependence of the four gene clusters on TcrA.

#### *wps*-1 gene cluster

The *wps*-1 gene cluster was activated at the early stage of sporangium formation (3 days of cultivation on HAT agar; Fig. S1C). The transcription of three genes adjacent to the *wps*-1 gene cluster (*AMIS_7260*, *AMIS_7270*, and *AMIS_7390*; Fig. S1A) was not activated on day 3 on HAT agar, indicating that these genes are under different transcriptional control (Fig. S1C). According to our previous comparative RNA-Seq analysis (25), transcription of the *wps*-1 gene cluster did not change significantly between the wild-type and Δ*tcrA* strains; the transcription of each gene was upregulated 1.4–3.0-fold in the Δ*tcrA* strain, except for *AMIS_7310*, whose transcript was 1.2-fold downregulated in the Δ*tcrA* strain. Therefore, we concluded that the transcription of the *wps*-1 gene cluster was not controlled by TcrA.

#### *wps*-2 gene cluster

Transcription of the *wps*-2 gene cluster was activated at the late stage of sporangium formation (6 days of cultivation on HAT agar), although transcriptional activation of *AMIS_56770* and *AMIS_56780* was rather weak (Fig. S2C). Transcription of the *wps*-2 gene cluster did not change significantly between the wild-type and Δ*tcrA* strains; the transcription of each gene was downregulated 1.3–1.9-fold in the Δ*tcrA* strain, except for *AMIS_56780*, whose transcript was 1.3-fold upregulated in the Δ*tcrA* strain (25), indicating that the transcription of the *wps*-2 gene cluster is not under the control of TcrA.

#### *wps*-3 gene cluster

Transcription of the *wps*-3 gene cluster was activated at the early stage of sporangium formation (3 days of cultivation on HAT agar; Fig. S3C). Transcription of the *wps*-3 gene cluster did not seem to be under the control of TcrA; the transcription of each gene was upregulated 2.3–4.1-fold in the Δ*tcrA* strain (25).

#### *gsf* gene cluster

Transcription of the *gsf* gene cluster was activated at the early stage of sporangium formation (3 days of cultivation on HAT agar; Fig. S4C). The transcriptional profiles of the five genes upstream from *AMIS_70480* (*AMIS_70490–AMIS_70530*; Fig. S4A) were different from those of the *gsf* gene cluster; transcription of *AMIS_70490*, *AMIS_70500*, and *AMIS_70510* was activated on day 1 on HAT agar, whereas transcription of *AMIS_70520* and *AMIS_70530* was not activated throughout the analyzed time course (Fig. S4C). Thus, these five genes seem to be under different transcriptional control from the *gsf* gene cluster. Transcription of the *gsf* gene cluster did not appear to be under the control of TcrA; the transcription of each gene was upregulated 1.4–2.7-fold in the Δ*tcrA* strain (25).

### The *wps*-1 and *gsf* gene clusters are involved in sporangium formation

**Fig 2:**
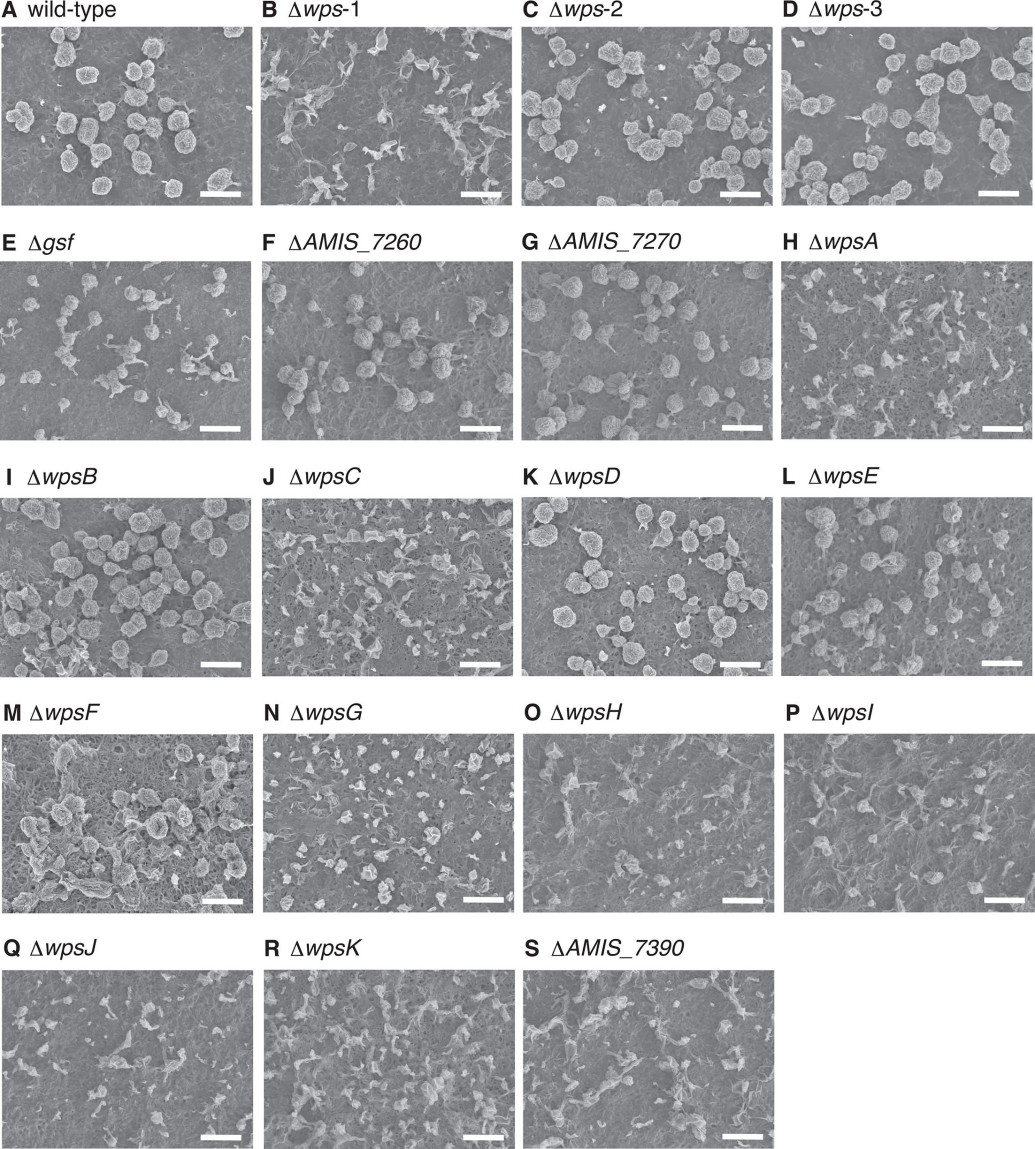
SEM observation of mycelia and sporangia formed on HAT agar after 7 days of cultivation. (**A**) Wild-type, (**B**) Δ*wps*-1, (**C**) Δ*wps*-2, (**D**) Δ*wps*-3, (**E**) Δ*gsf*, (**F**) Δ*AMIS_7260*, (**G**) Δ*AMIS_7270*, (**H**) Δ*wpsA*, (**I**) Δ*wpsB*, (**J**) Δ*wpsC*, (**K**) Δ*wpsD*, (**L**) Δ*wpsE*, (**M**) Δ*wpsF*, (**N**) Δ*wpsG*, (**O**) Δ*wpsH*, (**P**) Δ*wpsI*, (**Q**) Δ*wpsJ*, (**R**) Δ*wpsK*, and (**S**) Δ*AMIS_7390* strains are shown. Scale bars, 10 μm.

To examine the *in vivo* functions of the four gene clusters, we generated null mutant strains of each gene cluster (Δ*wps*-1, Δ*wps*-2, Δ*wps*-3, and Δ*gsf* strains). In the Δ*wps*-1, Δ*wps*-2, Δ*wps*-3, and Δ*gsf* strains, *wpsA–wpsK*, *AMIS_56690–AMIS_56780*, *AMIS_58090–AMIS_58170*, and *AMIS_70380–AMIS_70480*, respectively, were deleted from the chromosome of the wild-type strain (Fig. 1; Fig. S1A, S2A, S3A, and S4A). Macroscopic observation revealed no differences between the wild-type and four mutant strains grown on yeast extract-beef extract-NZ amine-maltose monohydrate (YBNM) and HAT agar. We observed mycelia and sporangia of the wild-type and mutant strains grown on HAT agar at 30°C for 7 days using scanning electron microscopy (SEM). The Δ*wps*-2 and Δ*wps*-3 strains produced normal globose and subglobose sporangia with short sporangiophores, similar to the wild-type sporangia, indicating that these gene clusters are not required for sporangium formation (Fig. 2A, C, and D). In contrast, severe defects were observed in the sporangia of the Δ*wps*-1 and Δ*gsf* strains; the Δ*wps*-1 strain produced sporangia in squashed shapes, and the Δ*gsf* strain generated sporangia smaller than the wild-type sporangia (Fig. 2B and E). These observations clearly indicate that the *wps*-1 and *gsf* gene clusters are involved in the formation of normal sporangia.

**Fig 3:**
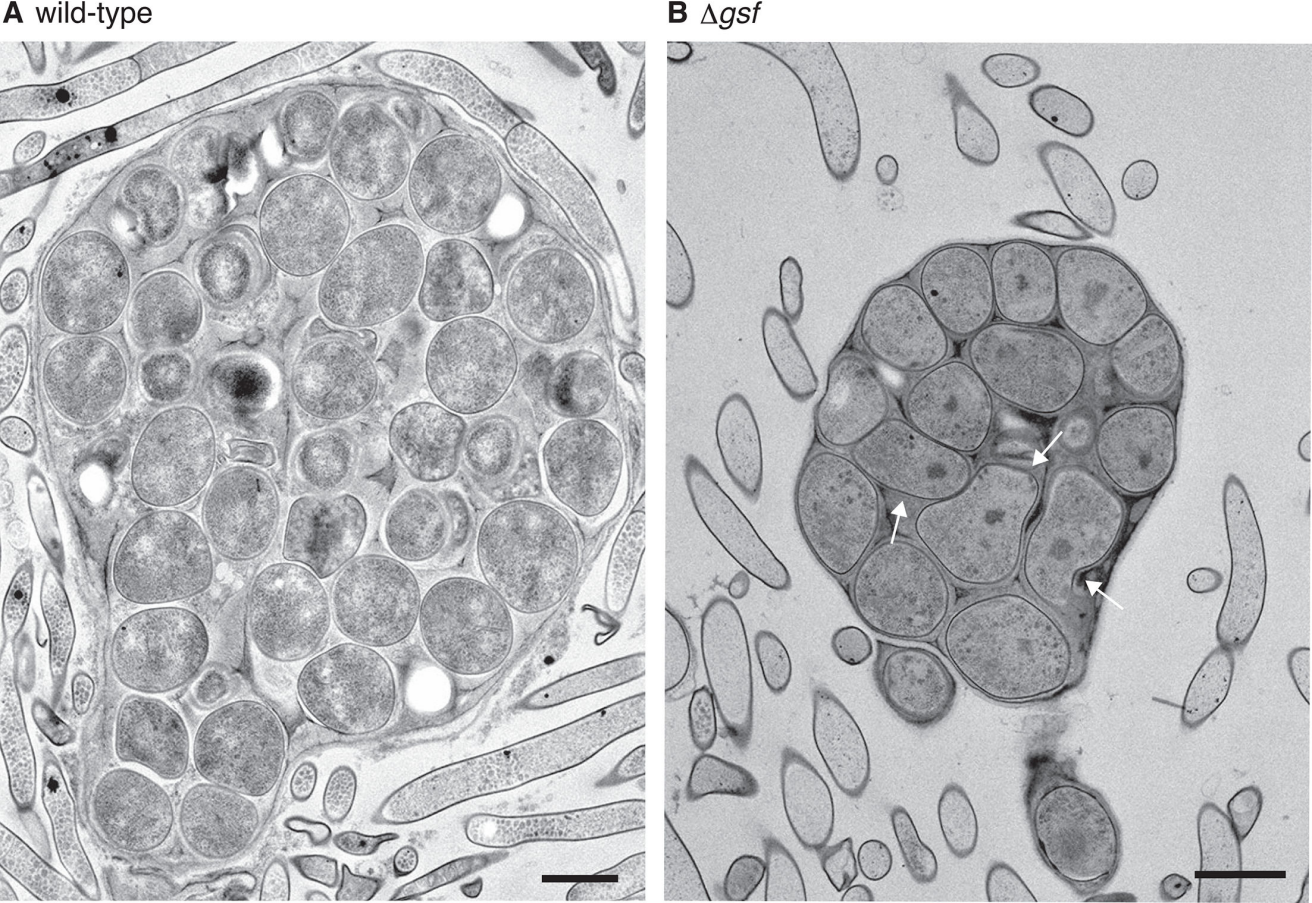
TEM analysis of ultrathin sections of sporangia produced on HAT agar. (**A**) Wild-type strain. (**B**) Δ*gsf* strain. In panel B, spores with distorted shapes are indicated by arrows. Bars, 1 μm.

We performed transmission electron microscopy (TEM) analysis of sporangia of the wild-type and Δ*gsf* strains grown under the same conditions for SEM analysis. As described in our previous study (26), the wild-type strain produced round spores of similar size, surrounded by the sporangium matrix (Fig. 3A). The Δ*gsf* strain also produced spores surrounded by the sporangium matrix. However, spore shapes were not uniform, and some spores were not round, indicating that the spores inside the Δ*gsf* sporangia were not fully matured (Fig. 3B).

**Fig 4:**
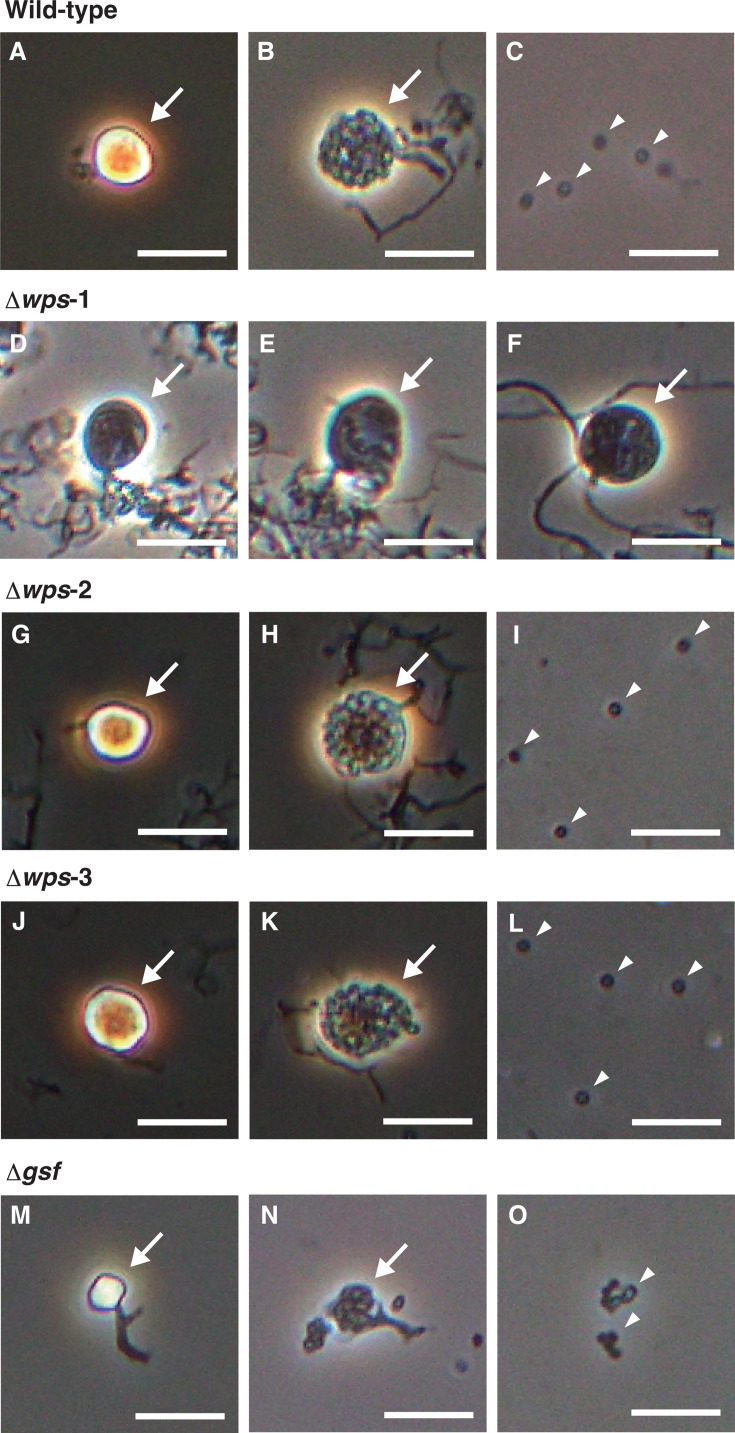
Observation of sporangium dehiscence using phase-contrast microscopy in the wild-type (**A–C**), Δ*wps*-1 (**D–F**), Δ*wps*-2 (**G–I**), Δ*wps*-3 (**J–L**), and Δ*gsf* (**M–O**) strains. Sporangia formed on HAT agar were harvested and suspended in 25 mM histidine solution to induce sporangium dehiscence. Panels **A, D, G, J,** and **M** show micrographs taken immediately after suspension. Panels **B, E, H, K,** and **N** show micrographs taken 15 min after suspension. Panels **C, F, I, L,** and **O** show micrographs taken 30 min after suspension. Sporangia (including transparent and immature sporangia) and zoospores are indicated by arrows and arrowheads, respectively. Scale bars, 5 μm.

We examined sporangium dehiscence using phase-contrast microscopy. Sporangium dehiscence can be induced by suspending the sporangia harvested from the agar surface in 25 mM histidine solution and incubating the suspension for 1 h. Under these conditions, the wild-type sporangia appear phase-bright immediately after suspension into histidine solution, and then the sporangium envelope gradually becomes transparent before spore release (Fig. 4A through C). Sporangium dehiscence proceeded normally in strains Δ*wps*-2 and Δ*wps*-3 (Fig. 4G–L). In the Δ*wps*-1 strain, sporangia appeared phase-dark immediately after suspension in histidine solution, and no spores were released throughout the time course of this observation (Fig. 4D through F). In the Δ*gsf* strain, the sporangia opened to release spores. However, spores of different sizes and spore chains were also observed in the Δ*gsf* strain (Fig. 4M through O).

**Fig 5:**
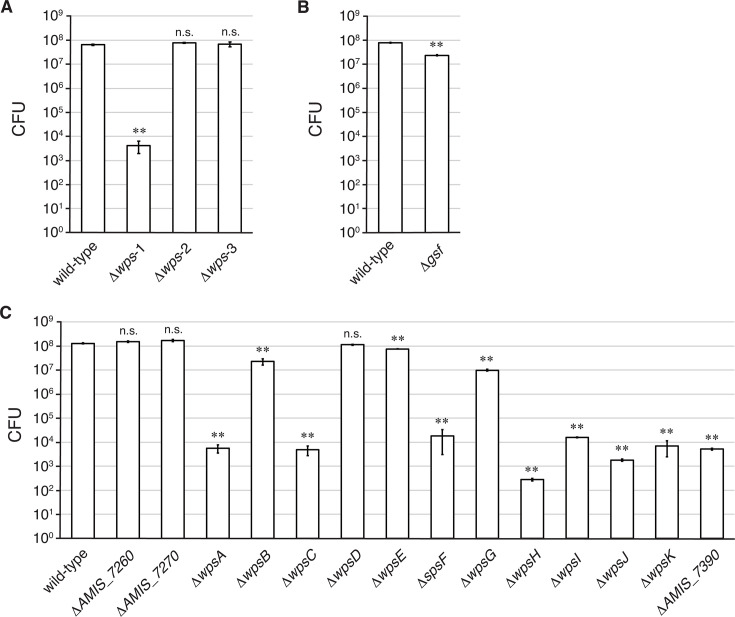
Number of spores released from the sporangia. Zoospores released from the sporangia by pouring 25 mM NH_4_HCO_3_ solution were counted as colony-forming units (CFU) on YBNM agar. (**A**) Wild-type, Δ*wps*-1, Δ*wps*-2, and Δ*wps*-3 strains. (**B**) Wild-type and Δ*gsf* strains. (**C**) Wild-type, Δ*AMIS_7260*, Δ*AMIS_7270*, Δ*wpsA*, Δ*wpsB*, Δ*wpsC*, Δ*wpsD*, Δ*wpsE*, Δ*wpsF*, Δ*wpsG*, Δ*wpsH*, Δ*wpsI*, Δ*wpsJ*, Δ*wpsK*, and Δ*AMIS_7390* strains. The values represent the mean ± standard error of at least three biological replicates. Differences were analyzed using Student’s *t*-test, and the levels of significance were set at ^**^P < 0.01. “n.s.” means no significant difference (*P* > 0.05). In panels A to C, the wild-type strain was used as a control, and the levels of significance for the wild-type strain are shown above each bar.

We quantified the spores released from the sporangia of the wild-type and mutant strains by counting the colonies formed on YBNM agar after incubation at 30°C for 2 days. The number of spores released from the sporangia of the Δ*wps*-2 and Δ*wps*-3 strains was similar to that released from the wild-type sporangia (Fig. 5A). In contrast, the number of spores released from the sporangia of the Δ*wps*-1 strain was significantly lower than that from the wild-type sporangia; the Δ*wps*-1 strain released only 10^3^ spores (per plate), while the wild-type strain released 10^7^ spores (Fig. 5A). The number of spores released from sporangia of the Δ*gsf* strain was also lower than that from the wild-type sporangia, although the number of spores of the Δ*gsf* strain was much higher than that of the Δ*wps*-1 strain (Fig. 5B). These results demonstrated that the *wps*-1 gene cluster is required for the formation of mature sporangia that can release spores under sporangium dehiscence-inducing conditions, whereas the *gsf* gene cluster is required for the formation of sporangia of normal size.

### Disruption of each gene in the *wps*-1 gene cluster

As described above, the *wps*-1 gene cluster contains genes that were predicted to encode a glycoside hydrolase (WpsE) and two sulfotransferases (WpsB and WpsH) in addition to a methyltransferase (WpsF) and protein kinase (WpsI) (Fig. S1B), suggesting that the gene products may produce a sulfated polysaccharide(s) with modifications. To examine the importance of such possible modifications, we generated null mutant strains of each gene in the *wps*-1 cluster (Δ*wpsA*–Δ*wpsK* strains). We also deleted each of the three genes adjacent to the *wps*-1 gene cluster (Δ*AMIS_7260*, Δ*AMIS_7270*, and Δ*AMIS_7390* strains). No differences were found between the wild-type and mutant strains grown on YBNM and HAT agar by macroscopic observation. We observed the mycelia and sporangia of the mutant strains grown on HAT agar at 30°C for 7 days using SEM. Normal sporangia similar to the wild-type sporangia were formed in the Δ*AMIS_7260*, Δ*AMIS_7270*, Δ*wpsB*, and Δ*wpsD* strains (Fig. 2F, G, I, and K). In contrast, abnormal squashed sporangia were observed in the Δ*wpsA*, Δ*wpsC*, Δ*wpsG*, Δ*wpsH*, Δ*wpsI*, Δ*wpsJ*, and Δ*wpsK* strains (Fig. 2H, J, and N through R). The sporangia of these mutants were similar to the sporangia of the Δ*wps*-1 strain (Fig. 2B). In the Δ*wpsE* strain, sporangia with abnormal shapes were observed in addition to normal sporangia (Fig. 2L). In the Δ*wpsF* strain, many distorted sporangia were connected to neighboring sporangia (Fig. 2M). Unexpectedly, severe defects in sporangium formation were observed in the Δ*AMIS_7390* strain (Fig. 2S).

Finally, we quantified the spores released from sporangia of strains with mutations within the *wps*-1 gene cluster under the same conditions as described above. The number of spores released from the sporangia of the Δ*AMIS_7260*, Δ*AMIS_7270*, and Δ*wpsD* strains was similar to that from the wild-type sporangia, which is consistent with normal sporangium formation in these strains (Fig. 5C). The number of spores released from the sporangia of the remaining mutant strains was significantly lower than that from the wild-type sporangia; sporangia of the Δ*wpsB* strain released only 10^7^ spores (per plate), whereas the wild-type sporangia released 10^8^ spores under the tested conditions (Fig. 5C). As described above, the Δ*wpsB* strain formed apparently normal sporangia when observed by SEM (Fig. 2I). Although Δ*wpsE* sporangia released nearly 10^8^ spores, the difference in the number of spores released between the wild-type and Δ*wpsE* sporangia was statistically significant (Fig. 5C). The Δ*wpsE* strain formed normal and abnormal sporangia, as observed by SEM (Fig. 2L). The sporangia of the remaining mutants (Δ*wpsA*, Δ*wpsC*, Δ*wpsF*, Δ*wpsG*, Δ*wpsH*, Δ*wpsI*, Δ*wpsJ*, Δ*wpsK*, and Δ*AMIS_7390* strains) released only 10^2^–10^6^ spores (Fig. 5C). Taken together, these results indicated that all genes in the *wps*-1 gene cluster, except *wpsD*, are required for normal sporangium formation.

## DISCUSSION

In this study, we characterized four gene clusters that were expected to be involved in EPS production and modification through Wzx/Wzy- and GtrA-dependent pathways by gene disruption. Severe defects in sporangium formation were observed in the Δ*wps*-1 and Δ*gsf* strains. Although no phenotypic changes were observed in the Δ*wps*-2 and Δ*wps*-3 strains under the conditions used in this study, we cannot exclude the possibility that these gene clusters are involved in the production of minor polysaccharide components in sporangia, considering the transcriptional activation of these gene clusters during sporangium formation. Because large chromosomal regions were deleted in the Δ*wps*-1, Δ*wps*-2, Δ*wps*-3, and Δ*gsf* strains (10, 11, 10, and 18 kbp, respectively), we were unable to conduct gene complementation tests in this study. However, it is very unlikely that the phenotypic changes observed in the Δ*wps*-1 and Δ*gsf* strains were derived from polar effects on the expression of neighboring genes, as they appear to belong to different transcriptional units from the genes of the respective gene cluster, based on the gene direction and their transcription profiles (Fig. S1 and S4).

**Fig 6:**
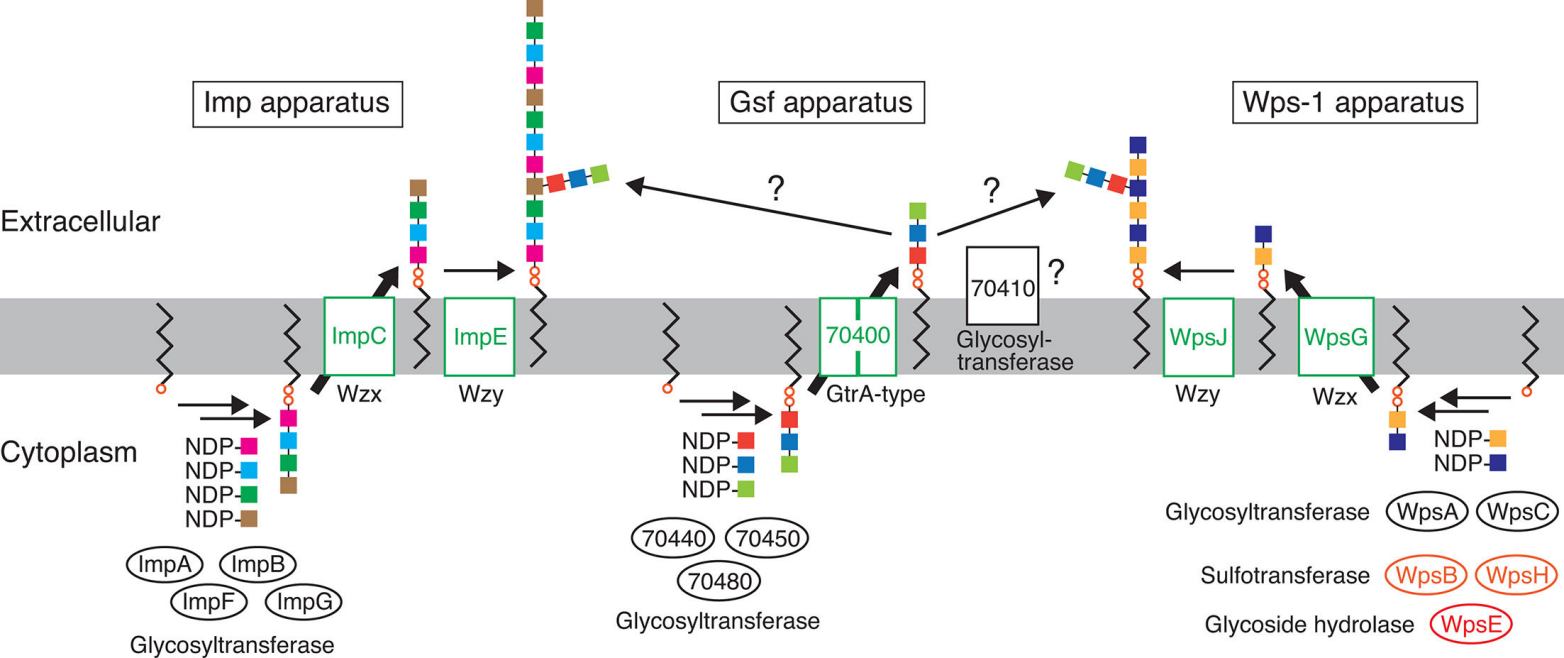
Schematic representation of a working hypothesis for sporangium matrix polysaccharide biosynthesis by the *imp*, *wps*-1, and *gsf* gene clusters. The *imp* and *wps*-1 gene clusters provide the Wxz/Wzy-dependent pathway, in which a repeating oligosaccharide unit is assembled by various glycosyltransferases in the first step. The stepwise assembly of the repeating unit occurs at the membrane-bound undecaprenol phosphate linker. For the Wps-1 apparatus, several modification enzymes catalyze the decoration of the oligosaccharide unit. Repeating oligosaccharides are translocated to the extracellular space by the Wzx protein, followed by polymerization by the Wzy protein. Wzz proteins are not shown. The *gsf* gene cluster provides a multi-component extracellular glycosylation system involving a GtrA-type flippase and four glycosyltransferases (three cytoplasmic and one transmembrane). The GtrA-type protein is likely to form a homodimer. The transmembrane glycosyltransferase AMIS_70410 may catalyze extracellular glycosylation, although AMIS_70410 is not a GT-C-fold glycosyltransferase. In this hypothetical model, oligosaccharides produced by the Imp, Wps-1, and Gsf machinery consist of four, two, and three sugars, respectively, based on the number of glycosyltransferases encoded in each gene cluster. However, the number of sugars consisting of repeating oligosaccharides and the length of the polysaccharide strands remain unknown at this stage. It should be noted that the biosynthetic pathways presented in this hypothetical model have not yet been experimentally demonstrated.

As described in the Introduction, we recently reported that the *imp* gene cluster plays a pivotal role in sporangium matrix polysaccharide production (18); we generated and analyzed null mutants of each gene in the *imp* cluster (Δ*impA*–Δ*impG* strains) and the null mutant of the whole *imp* cluster (Δ*imp* cluster strain; Fig. 1). It was a big mystery that the deficiency of sporangium formation observed in the null mutants of each gene, except for Δ*impG*, was much more severe than that in the Δ*imp* cluster strain (18). In the Δ*imp* cluster strain, sporangia with a normal, round shape were abundantly produced, and the number of spores released from sporangia was reduced only slightly (18). However, the sporangium matrix of the Δ*imp* cluster strain appeared to be different from that of the wild-type strain, as observed using TEM; it was more electron-dense (18). We speculate that this sporangium matrix substance is a polysaccharide synthesized by the *wps*-1 and *gsf* gene clusters. We also speculate that the partial loss of the Imp machinery has more serious effects on sporangium matrix polysaccharide biosynthesis than the complete loss because the complete loss of the Imp machinery may provide abundant NDP-sugars and/or undecaprenol phosphate anchors to the Wps-1 and Gsf apparatus, resulting in the overproduction of EPSs produced by the Wps-1 and Gsf machinery inside sporangia. Furthermore, it is worth noting that the following phenotypic changes in the Δ*impD* and Δ*wps*-1 strains were similar to each other: (i) both strains formed abnormal sporangia in squashed shapes, (ii) sporangia of both strains appeared phase-dark immediately after suspension in histidine solution, and (iii) sporangia of the Δ*impD* and Δ*wps*-1 strains released only 10^2^ and 10^3^ spores (per plate), respectively, under sporangium dehiscence-inducing conditions (Fig. 2B; Fig. 4D through F; Fig. 5A) (18). Notably, no sporangium matrix was observed inside the Δ*impD* sporangium using TEM (18). ImpD is a functionally unknown protein with five transmembrane helices. In the *imp* cluster, *impC* and *impE* encode membrane proteins that presumably function as Wzx and Wzy proteins, respectively, based on their amino acid sequence similarities. Therefore, we speculated that ImpD may assist the functions of ImpC and/or ImpE or function as a Wzz protein that controls the length of the produced polysaccharide. Taken together, we considered that the *wps*-1 gene cluster is involved in sporangium matrix polysaccharide biosynthesis, along with the *imp* gene cluster. Thus, sporangium matrix polysaccharides may be more complex than previously expected. Not only polysaccharides synthesized by the *imp* cluster, but also different polysaccharides synthesized by the *wps*-1 cluster and their modification by oligosaccharides synthesized by the *gsf* cluster seem to be essential for the biogenesis of the intact sporangium matrix. Based on this information, we propose a working hypothesis for sporangium matrix polysaccharide biosynthesis involving the *imp*, *wps*-1, and *gsf* gene clusters in *A. missouriensis* (Fig. 6). Because most pathways in this hypothetical model have not been demonstrated experimentally, the chemical structures of sporangium matrix polysaccharides and the contribution of each gene cluster are crucial subjects for future research.

According to the CAZy database, four glycosyltransferases encoded in the *wps*-1 and *gsf* gene clusters (WpsA, AMIS_70410, AMIS_70450, and AMIS_70480) belong to the GT2 family. Members of this family have a GT-A fold, which consists of two dissimilar domains: one involved in binding to NDP sugars and the other in binding to sugar acceptors (27). Thus, we constructed a phylogenetic tree using WpsA, AMIS_70410, AMIS_70450, AMIS_70480, ImpG, and 94 functionally characterized glycosyltransferases from the GT2 family. ImpG is a GT2 family enzyme encoded by the *imp* gene cluster (18). WpsA formed a clade containing two galactofuranosyltransferases, whereas AMIS_70410 was in a clade containing a glucosyltransferase, an *N*-acetylglucosaminyltransferase, and an *N*-acetylgalactosaminyltransferase. AMIS_70450 and AMIS_70480 were found to belong to a clade containing a glucosyltransferase (Fig. S5A). We also constructed a phylogenetic tree using WpsC and five functionally characterized glycosyltransferases from the GT26 family. WpsC formed a clade with a glucosyltransferase (Fig. S5B). These phylogenetic analyses suggest that a repeat unit of the polysaccharide produced by the *wps*-1 gene cluster is a heterooligosaccharide, whose sugar moieties may be composed of galactofuranose and glucose, while the gene products of the *gsf* gene cluster may be involved in the glycosylation of target compounds with oligosaccharides composed of glucose, *N*-acetylglucosamine, and/or *N*-acetylgalactosamine.

In the gram-negative bacterial pathogen *Shigella flexneri*, several mutant strains that have a deficiency in the Wzx/Wzy-dependent O-antigen oligosaccharide biosynthetic pathway are lethal when they accumulate a large amount of undecaprenol diphosphate-linked O-antigen intermediates on the cytoplasmic side of the cellular membrane (28, 29). Examples include mutants that lack (i) late glycosyltransferases in the polysaccharide assembly pathway (28), (ii) Wzx flippase under forced expression of *wecA*, which encodes the initial glycosyltransferase (28), and (iii) Wzy polymerase (29). Undecaprenol phosphate, which is recycled in the O-antigen oligosaccharide biosynthetic pathway, is also essential for the biosynthesis of peptidoglycan and other cell envelope components. Therefore, the sequestration of undecaprenol phosphate at the cytosolic side of the inner membrane leads to growth defects (30). In contrast, all mutant strains of the genes in the *wps*-1 gene cluster grew normally in *A. missouriensis*. The *wps*-1 gene cluster appears to be expressed only in the maturing spores and/or hypha extending in sporangia during sporangium formation. Therefore, it is reasonable to assume that disturbance of Wps-1-dependent polysaccharide biosynthesis has no effect on mycelial growth. However, the accumulation of undecaprenol diphosphate-linked intermediates at the cytosolic face of the cytoplasmic membrane likely occurs in maturing spores and/or hyphae inside the sporangia of some single-gene disruptants. The possible sequestration of undecaprenol diphosphate-linked intermediates in such mutant sporangia may account for the deficiency in sporangium development. Notably, all mutant strains of the genes in the *imp* gene cluster also grew normally, although most of them were deficient in sporangium development (18), as described in the second paragraph of the Discussion section.

In the *wps*-1 gene cluster, *wpsB* and *wpsH*, both of which are required for normal sporangium formation, were predicted to encode a sulfotransferase, thereby raising the possibility that sulfated polysaccharide(s) are a component of the sporangium matrix. As described in the Introduction, bacterial sulfated polysaccharides are exclusively produced in cyanobacteria. We believe that the characterization of the *wps*-1 gene cluster conducted in this study is significant because it suggests that sulfated polysaccharides are produced in phylogenetically distant actinomycetes. However, the production of sulfated polysaccharides in *A. missouriensis* awaits experimental verification. In this gene cluster, *wpsE* was predicted to encode a putative GH26 family enzyme. Enzymes in this family primarily function as endo-β−1,4-mannanases, although an exo-β-mannanase has also been reported (31, 32). To the best of our knowledge, no gene cluster for the Wzx/Wzy-dependent pathway involving a GH26 enzyme has been characterized. Unexpectedly, the Δ*AMIS_7390* strain showed a severe defect in sporangium formation. *AMIS_7390* was predicted to encode a member of the sulfate adenylyltransferase and adenosine 5′-phosphosulfate (APS) kinase family, which catalyzes the formation of APS from inorganic sulfate and ATP at a key step in the biosynthesis of sulfur-containing amino acids (33). To the best of our knowledge, the relationship between the members of this enzyme family and morphological development has not yet been reported.

Although defects in sporangium formation were observed in the Δ*gsf* strain, the phenotypic changes observed in this mutant were less severe than those in the Δ*wps*-1 strain. As described in the Introduction, GtrA-type protein-containing glycosylation systems are involved in the glycosylation of cell wall polymers such as LTAs, WTAs, and SCWPs in gram-positive bacteria (5). We assume that the *gsf* gene cluster may be responsible for the modification of sporangium matrix polysaccharides in *A. missouriensis*. Considering that smaller sporangia were observed in the Δ*gsf* strain, this potential modification of the sporangium matrix polysaccharides may be important for the expansion of sporangia. This suggests that the production of normal sporangium matrix polysaccharides is essential for the production of sporangia with a normal shape and spore maturation inside the sporangia. Although it is very difficult to prepare a sufficient amount of sporangium matrix polysaccharides for analysis, future investigations of the *wps*-1 and *gsf* gene clusters, as well as the *imp* gene cluster, will provide clues to the chemical structures of sporangium matrix polysaccharides and the molecular mechanisms of sporangium formation in *A. missouriensis*.

## MATERIALS AND METHODS

### General methods

Bacterial strains, plasmid vectors, and media used in this study have been described previously (34–36). Primers used in this study are listed in Table S1. SEM was performed using an S-4800 scanning electron microscope (Hitachi, Tokyo, Japan), as described previously (37). Ultrathin sections of sporangia were observed using a JEM-1400Plus transmission electron microscope (Jeol, Tokyo, Japan) by Tokai Electron Microscopy, Inc. (Aichi, Japan). Phase-contrast microscopic observations were performed using a BH-2 microscope (Olympus, Tokyo, Japan), as described previously (38). Free zoospores were quantified as described previously (39).

### Construction of gene deletion mutants

To construct gene deletion mutant strains, the upstream and downstream regions of the target genes or gene clusters were amplified using PCR. The amplified fragments were digested with restriction enzymes (Table S1) and cloned into pUC19 digested with the same restriction enzymes. The generated plasmids were sequenced to confirm the absence of any errors. The cloned fragments were digested with restriction enzymes and cloned together into pK19mobsacB (40), whose kanamycin resistance gene had been replaced with the apramycin resistance gene *aac(3)IV*, digested with restriction enzymes. The generated plasmids were introduced into the *A. missouriensis* wild-type strain by conjugation, as described previously (41). Apramycin-resistant colonies generated by single-crossover recombination were isolated. One of them was cultivated in peptone-yeast extract-magnesium (PYM) liquid broth at 30°C for 36–48 h, and the mycelia suspended in 0.75% NaCl solution were spread onto Czapek-Dox broth (BD, NJ, USA) agar medium containing extra sucrose (final concentration of 5%). After incubation at 30°C for 5 days, sucrose-resistant colonies were inoculated onto YBNM agar medium with or without apramycin to confirm that they were sensitive to apramycin. Apramycin-sensitive and sucrose-resistant colonies generated by the second single-crossover recombination were isolated as candidates for gene deletion mutants. Disruption of the target genes or gene clusters was confirmed using PCR.

## Data Availability

The data underlying this article are available in the article and in its supplemental material.

## SUPPLEMENTAL MATERIAL

Supplemental material aem.02330-25-s0001.pdf Fig. S1 to S5; Table S1.

ASM does not own the copyrights to Supplemental Material that may be linked to, or accessed through, an article. The authors have granted ASM a non-exclusive, world-wide license to publish the Supplemental Material files. Please contact the corresponding author directly for reuse.
