# Supplementary material for: Involvement of two putative gene clusters for exopolysaccharide biosynthesis and modification in sporangium formation in Actinoplanes missouriensis
Source: Appl Environ Microbiol. 2026 Jun 11;92(7):e02330-25. doi: 10.1128/aem.02330-25 (PMC13390465; doi:10.1128/aem.02330-25)
Supplement: Supplemental material — Fig. S1 to S5; Table S1. [file aem.02330-25-s0001.pdf]

Supporting Information for

Involvement of two putative gene clusters for exopolysaccharide biosynthesis and modification in sporangium formation in *Actinoplanes missouriensis*

Takeaki Tezuka,[1,2] Yasuo Ohnishi[1,2]

[1]Department of Biotechnology, Graduate School of Agricultural and Life Sciences, The University of Tokyo, Bunkyo-ku, Tokyo, Japan

[2]Collaborative Research Institute for Innovative Microbiology, The University of Tokyo, Bunkyo-ku, Tokyo, Japan

This PDF file includes:

Figures S1 to S5
Table S1

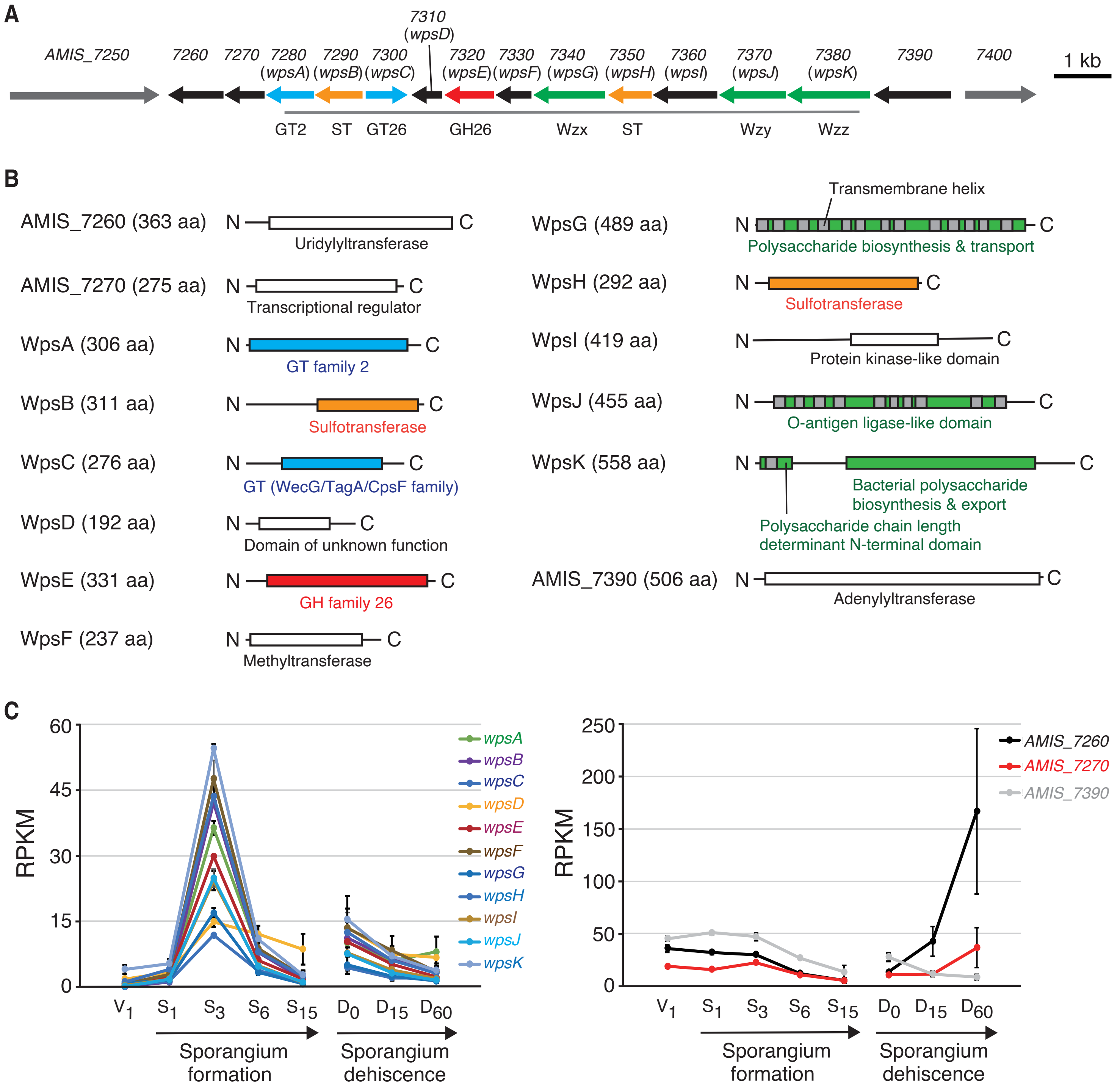

Fig. S1. Gene organization and transcriptional profile of the *wps*-1 gene cluster and domain organization of the gene products. (A) Gene organization around the *wps*-1 gene cluster. Arrows indicate the locations of the open reading frames, including their lengths and directions. Genes encoding glycosyltransferases are colored light blue. Genes encoding the Wzx/Wzy-dependent pathway are shown in green. Sulfotransferase and glycosyl hydrolase genes are colored orange and red, respectively. Gene identification numbers are shown above the arrows. Gene names are shown in parentheses. The deleted region in the Δ*wps*-1 strain is shown by a gray rectangle below the arrows. (B) Domain organization of the gene products of the *wps*-1 gene cluster and three adjacent genes (*AMIS_7260*, *AMIS_7270*, and *AMIS_7390*). (C) Transcript levels of the 11 genes comprising the *wps*-1 gene cluster and the three adjacent genes. Transcripts were examined using RNA-Seq analysis under various culture conditions. RNA samples were prepared from substrate hyphae grown on YBNM agar for 1 day (V_1), substrate hyphae or mixtures of substrate hyphae and sporangia grown on HAT agar for 1, 3, 6, and 15 days (S_1, S_3, S_6, and S_{15}, respectively), and sporangia (including some substrate hyphae) incubated in 25 mM histidine solution to induce sporangium dehiscence for 0, 15, and 60 min (D_0, D_{15}, and D_{60}, respectively). The values are the average number of reads per kilobase of coding sequence per million mapped reads (RPKM) ± standard error of three biological replicates.

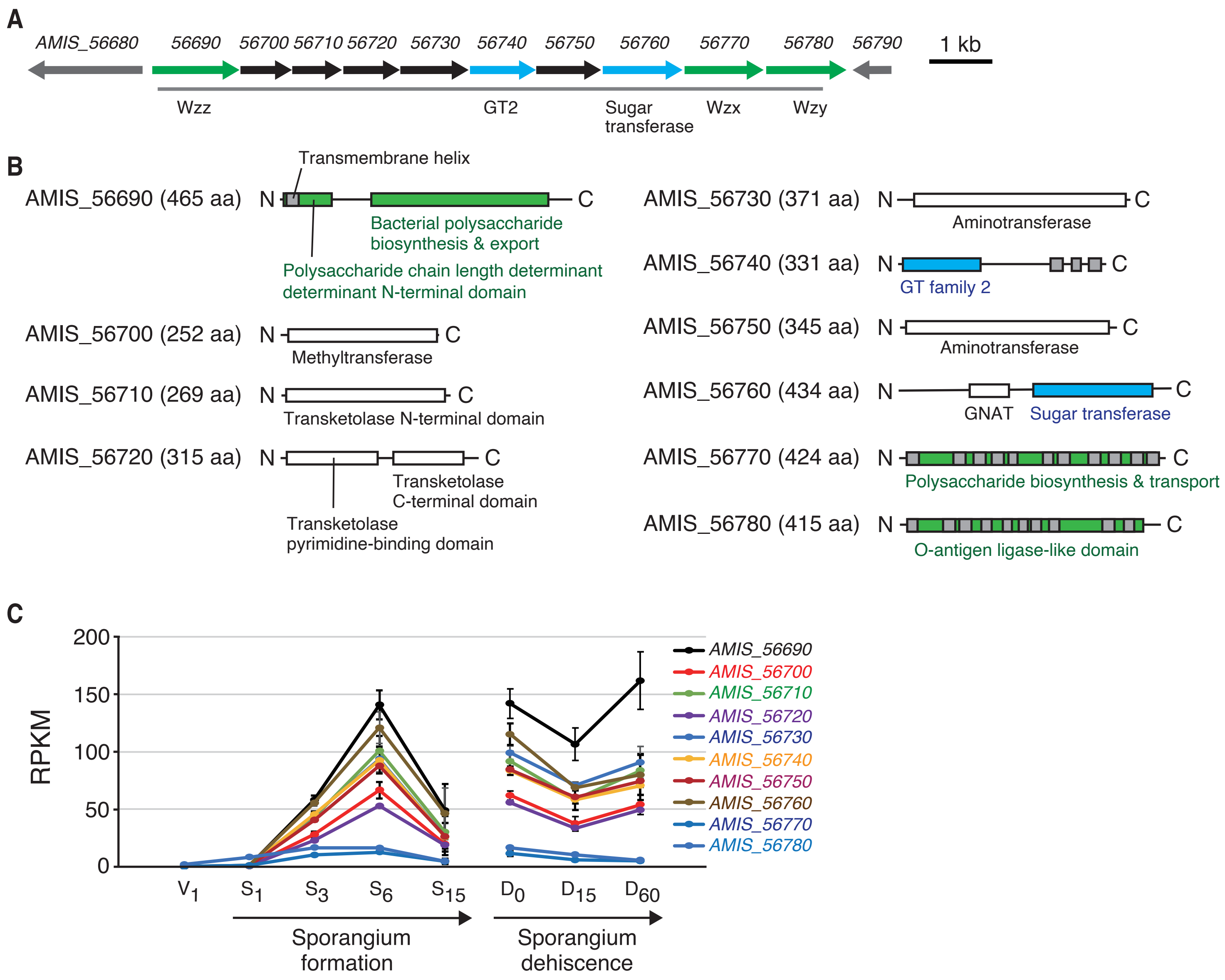

Fig. S2. Gene organization and transcriptional profile of the *wps*-2 gene cluster and domain organization of the gene products. (A) Gene organization around the *wps*-2 gene cluster. Arrows indicate the locations of the open reading frames, including their lengths and directions. Genes encoding glycosyltransferases (or sugar transferases) are colored light blue. Genes encoding the Wzx/Wzy-dependent pathway are shown in green. Gene identification numbers are shown above the arrows. The deleted region in the Δ*wps*-2 strain is shown by a gray rectangle below the arrows. (B) Domain organization of the gene products of the *wps*-2 gene cluster. (C) Transcript levels of the ten genes comprising the *wps*-2 gene cluster. Transcripts were examined as described in the legend of Fig. S1.

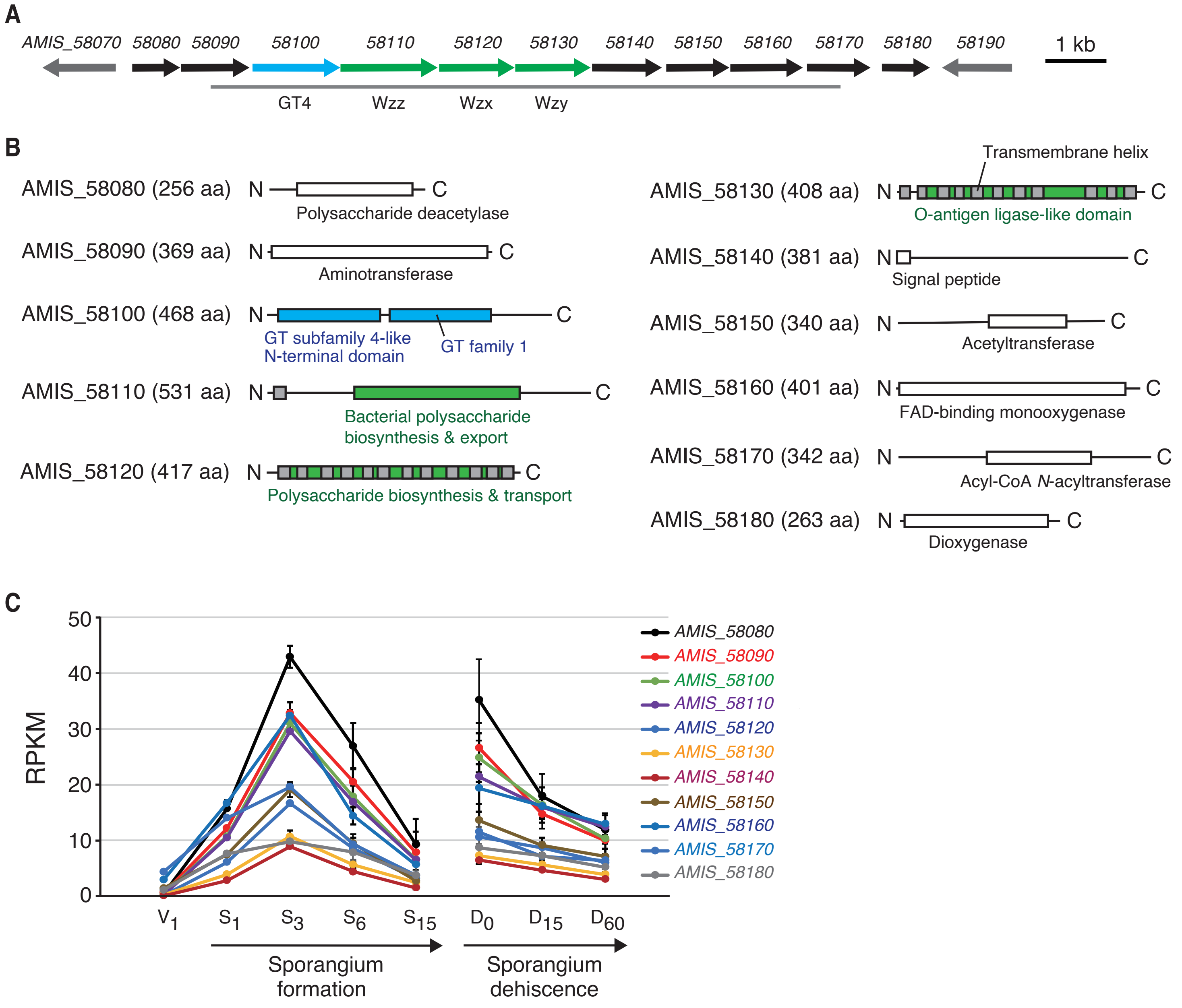

Fig. S3. Gene organization and transcriptional profile of the *wps*-3 gene cluster and domain organization of the gene products. (A) Gene organization around the *wps*-3 gene cluster. Arrows indicate the locations of the open reading frames, including their lengths and directions. A glycosyltransferase gene is colored light blue. Genes encoding the Wzx/Wzy-dependent pathway are shown in green. Gene identification numbers are shown above the arrows. The deleted region in the Δ*wps*-3 strain is shown by a gray rectangle below the arrows. (B) Domain organization of the gene products of the *wps*-3 gene cluster. (C) Transcript levels of the 11 genes comprising the *wps*-3 gene cluster. Transcripts were examined as described in the legend of Fig. S1.

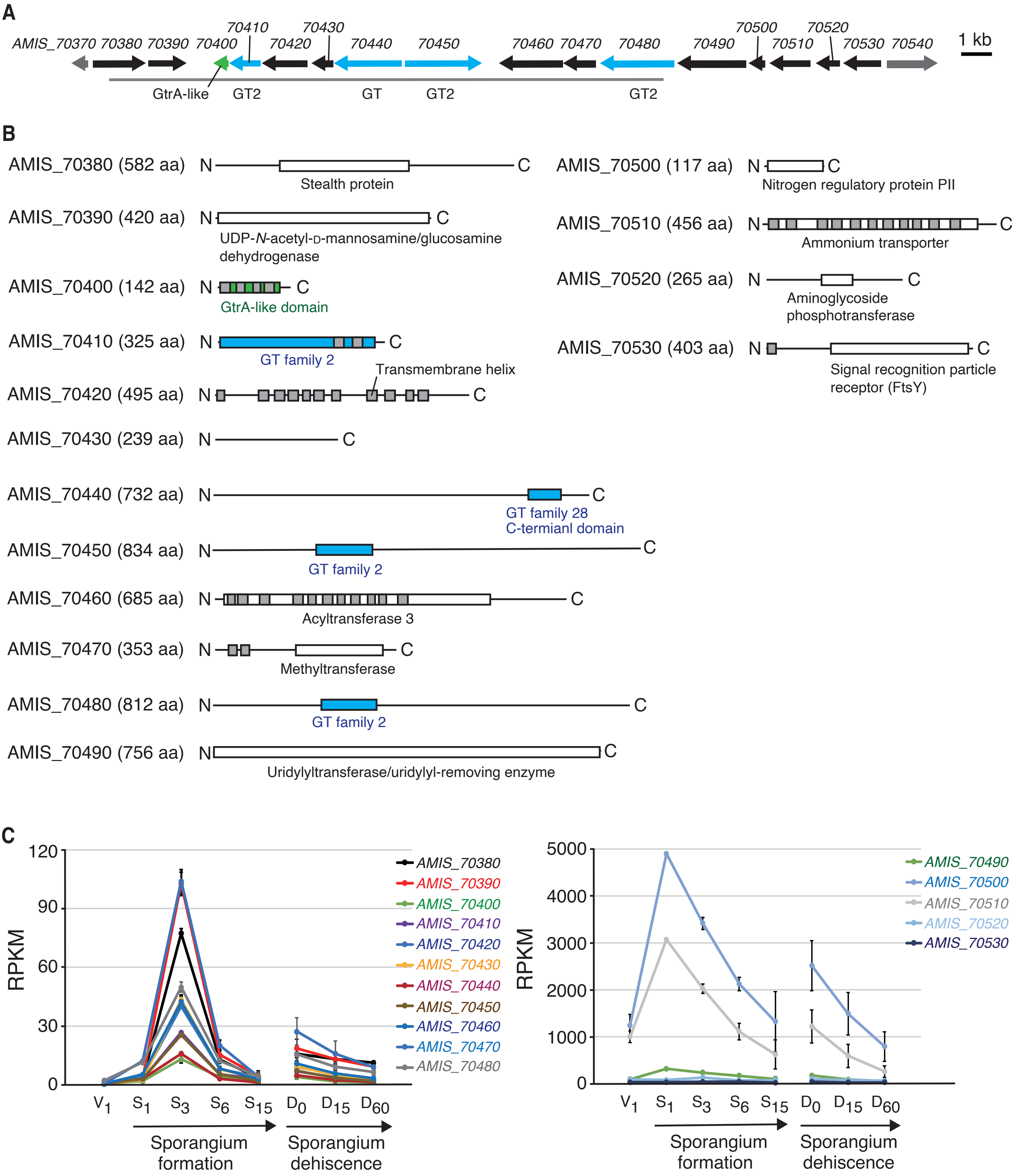

Fig. S4. Gene organization and transcriptional profile of the *gsf* gene cluster and domain organization of the gene products. (A) Gene organization around the *gsf* gene cluster. Arrows indicate the locations of the open reading frames, including their lengths and directions. Genes encoding glycosyltransferases are colored light blue. A gene encoding a GtrA-like protein is colored green. Gene identification numbers are shown above the arrows. The deleted region in the Δ*gsf* strain is shown by a gray rectangle below the arrows. (B) Domain organization of the gene products of the *gsf* gene cluster and five adjacent genes (*AMIS_70490*, *AMIS_70500*, *AMIS_70510*, *AMIS_70520*, and *AMIS_70530*). (C) Transcript levels of the 11 genes comprising the *gsf* gene cluster and the five adjacent genes. Transcripts were examined as described in the legend of Fig. S1.

A

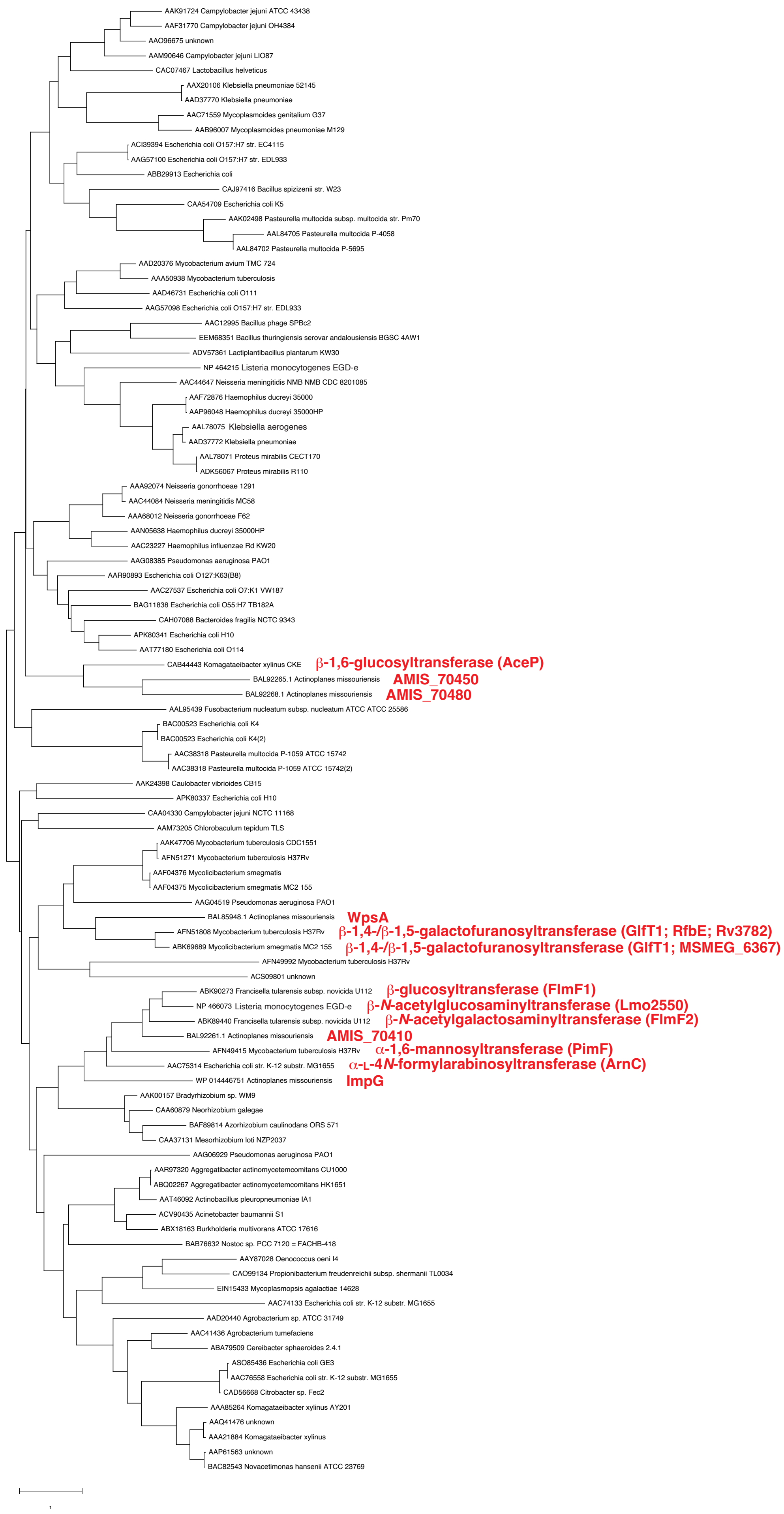
AAK91724 Campylobacter jejuni ATCC 43438
AAF31770 Campylobacter jejuni OH4384
AAO96675 unknown
AAM90646 Campylobacter jejuni LIO87
CAC07467 Lactobacillus helveticus
AAX20106 Klebsiella pneumoniae 52145
AAD37770 Klebsiella pneumoniae
AAC71559 Mycoplasmoides genitalium G37
AAB96007 Mycoplasmoides pneumoniae M129
ACI39394 Escherichia coli O157:H7 str. EC4115
AAG57100 Escherichia coli O157:H7 str. EDL933
ABB29913 Escherichia coli
CAJ97416 Bacillus spizizenii str. W23
CAA54709 Escherichia coli K5
AAK02498 Pasteurella multocida subsp. multocida str. Pm70
AAL84705 Pasteurella multocida P-4058
AAL84702 Pasteurella multocida P-5695
AAD20376 Mycobacterium avium TMC 724
AAA50938 Mycobacterium tuberculosis
AAD46731 Escherichia coli O111
AAG57098 Escherichia coli O157:H7 str. EDL933
AAC12995 Bacillus phage SPBc2
EEM68351 Bacillus thuringiensis serovar andalousiensis BGSC 4AW1
ADV57361 Lactiplantibacillus plantarum KW30
NP 464215 Listeria monocytogenes EGD-e
AAC44647 Neisseria meningitidis NMB NMB CDC 8201085
AAF72876 Haemophilus ducreyi 35000
AAP96048 Haemophilus ducreyi 35000HP
AAL78075 Klebsiella aerogenes
AAD37772 Klebsiella pneumoniae
AAL78071 Proteus mirabilis CECT170
ADK56067 Proteus mirabilis R110
AAA92074 Neisseria gonorrhoeae 1291
AAC44084 Neisseria meningitidis MC58
AAA68012 Neisseria gonorrhoeae F62
AAN05638 Haemophilus ducreyi 35000HP
AAC23227 Haemophilus influenzae Rd KW20
AAG08385 Pseudomonas aeruginosa PAO1
AAR90893 Escherichia coli O127:K63(B8)
AAC27537 Escherichia coli O7:K1 VW187
BAG11838 Escherichia coli O55:H7 TB182A
CAH07088 Bacteroides fragilis NCTC 9343
APK80341 Escherichia coli H10
AAT77180 Escherichia coli O114
CAB44443 Komagataeibacter xylinus CKE β-1,6-glucosyltransferase (AceP)
BAL92265.1 Actinoplanes missouriensis AMIS_70450
BAL92268.1 Actinoplanes missouriensis AMIS_70480
AAL95439 Fusobacterium nucleatum subsp. nucleatum ATCC ATCC 25586
BAC00523 Escherichia coli K4
BAC00523 Escherichia coli K4(2)
AAC38318 Pasteurella multocida P-1059 ATCC 15742
AAC38318 Pasteurella multocida P-1059 ATCC 15742(2)
AAK24398 Caulobacter vibrioides CB15
APK80337 Escherichia coli H10
CAA04330 Campylobacter jejuni NCTC 11168
AAM73205 Chlorobaculum tepidum TLS
AAK47706 Mycobacterium tuberculosis CDC1551
AFN51271 Mycobacterium tuberculosis H37Rv
AAF04376 Mycolicibacterium smegmatis
AAF04375 Mycolicibacterium smegmatis MC2 155
AAG04519 Pseudomonas aeruginosa PAO1
BAL85948.1 Actinoplanes missouriensis WpsA
AFN51808 Mycobacterium tuberculosis H37Rv β-1,4-/β-1,5-galactofuranosyltransferase (GlfT1; RfbE; Rv3782)
ABK69689 Mycolicibacterium smegmatis MC2 155 β-1,4-/β-1,5-galactofuranosyltransferase (GlfT1; MSMEG_6367)
AFN49992 Mycobacterium tuberculosis H37Rv
ACS09801 unknown
ABK90273 Francisella tularensis subsp. novicida U112 β-glucosyltransferase (FlmF1)
NP 466073 Listeria monocytogenes EGD-e β-N-acetylglucosaminyltransferase (Lmo2550)
ABK89440 Francisella tularensis subsp. novicida U112 β-N-acetylgalactosaminyltransferase (FlmF2)
BAL92261.1 Actinoplanes missouriensis AMIS_70410
AFN49415 Mycobacterium tuberculosis H37Rv α-1,6-mannosyltransferase (PimF)
AAC75314 Escherichia coli str. K-12 substr. MG1655 α-L-4N-formylarabinosyltransferase (ArnC)
WP 014446751 Actinoplanes missouriensis ImpG
AAK00157 Bradyrhizobium sp. WM9
CAA60879 Neorhizobium galegae
BAF89814 Azorhizobium caulinodans ORS 571
CAA37131 Mesorhizobium loti NZP2037
AAG06929 Pseudomonas aeruginosa PAO1
AAR97320 Aggregatibacter actinomycetemcomitans CU1000
ABQ02267 Aggregatibacter actinomycetemcomitans HK1651
AAT46092 Actinobacillus pleuropneumoniae IA1
ACV90435 Acinetobacter baumannii S1
ABX18163 Burkholderia multivorans ATCC 17616
BAB76632 Nostoc sp. PCC 7120 = FACHB-418
AAY87028 Oenococcus oeni I4
CAO99134 Propionibacterium freudenreichii subsp. shermanii TL0034
EIN15433 Mycoplasmopsis agalactiae 14628
AAC74133 Escherichia coli str. K-12 substr. MG1655
AAD20440 Agrobacterium sp. ATCC 31749
AAC41436 Agrobacterium tumefaciens
ABA79509 Cereibacter sphaeroides 2.4.1
ASO85436 Escherichia coli GE3
AAC76558 Escherichia coli str. K-12 substr. MG1655
CAD56668 Citrobacter sp. Fec2
AAA85264 Komagataeibacter xylinus AY201
AAQ41476 unknown
AAA21884 Komagataeibacter xylinus
AAP61563 unknown
BAC82543 Novacetimonas hansenii ATCC 23769
1

B

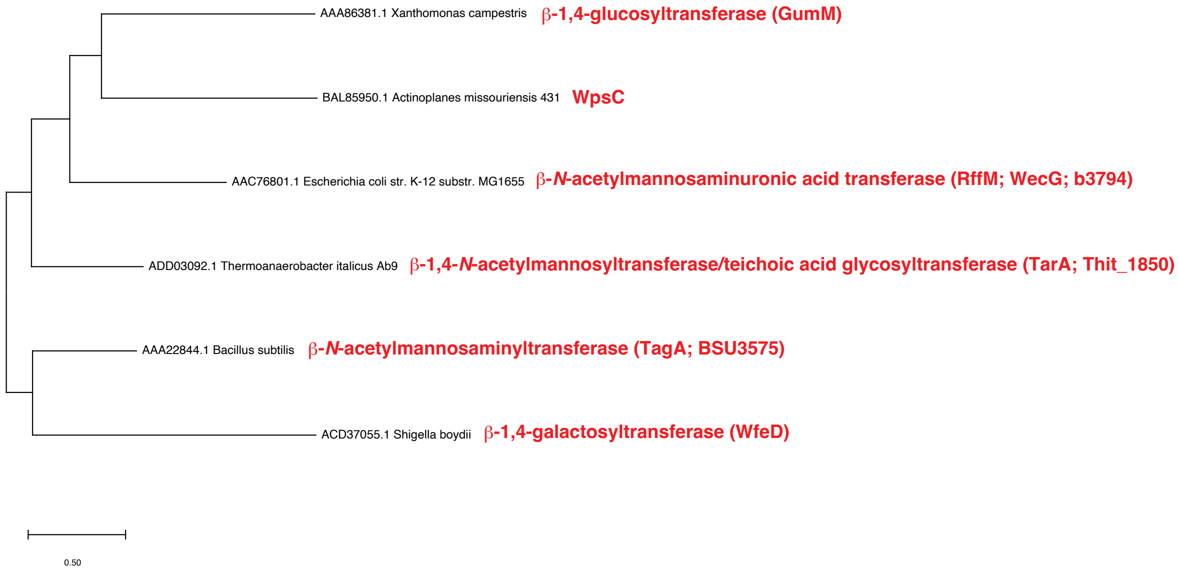

Fig. S5. Phylogenetic analysis of glycosyltransferases. Maxumum likelihood trees of 99 glycosyltransferases from the GT2 family (A) and 6 glycosyltransferases from the GT26 family (B) were constructed using MEGA X. WpsA, WpsC, AMIS_70410, AMIS_70450, AMIS_70480, and several characterized enzymes are shown in red.

Table S1. Primers used in this study

Primer name	Sequence (5' to 3')[a]	Restriction enzyme	Used for
Wzy-III-UF2	GCCAAGCTTCCGGTTCTGTCTCACAAACA	HindIII	Disruption of the *wps*-1 gene cluster
Wzy-III-UR2	GCTCTAGAGTACACCTTGGGCATGGTCT	XbaI	Disruption of the *wps*-1 gene cluster
Wzy-III-DF2	GCTCTAGAGTGAAGGTGGAGAACGTGCT	XbaI	Disruption of the *wps*-1 gene cluster
Wzy-III-DR2	GGAATTCGGGACCACATCGTTGACGAA	EcoRI	Disruption of the *wps*-1 gene cluster
AMIS_56690-56780-UF1	GGAATTCAGCTCACCGAACGGCGGAAT	EcoRI	Disruption of the *wps*-2 gene cluster
AMIS_56690-56780-UR1	GCTCTAGACACCAGCCACCAGTGTCTTT	XbaI	Disruption of the *wps*-2 gene cluster
AMIS_56690-56780-DF1	GCTCTAGACTCTTCTCCGGCGACTACTT	XbaI	Disruption of the *wps*-2 gene cluster
AMIS_56690-56780-DR1	GCCAAGCTTACTTCACGCTGCTCTGGAGT	HindIII	Disruption of the *wps*-2 gene cluster
AMIS_58090-58170-UF1	CGTAGAATTCGTCGGCGAAT	EcoRI	Disruption of the *wps*-3 gene cluster
AMIS_58090-58170-UR1	GCTCTAGAGATGATCTTGTTGCCGTTGA	XbaI	Disruption of the *wps*-3 gene cluster
AMIS_58090-58170-DF1	GCTCTAGACGTTCTACTTCTTCGAGCGT	XbaI	Disruption of the *wps*-3 gene cluster
AMIS_58090-58170-DR1	GCCAAGCTTGTCATCGTCGGTGACCTCAA	HindIII	Disruption of the *wps*-3 gene cluster
AMIS_70380-UF1	GGAATTCTCGAGTTTCGGCAGCTTGAA	EcoRI	Disruption of the *gsf* gene cluster
AMIS_70380-UR1	GCTCTAGATGTCGCGATCGTTCTCGGTCA	XbaI	Disruption of the *gsf* gene cluster
AMIS_70480-UF1	GCTCTAGAGTCGTCCACGATCAGGATCT	XbaI	Disruption of the *gsf* gene cluster
AMIS_70480-UR1	GCCAAGCTTCCTGCTCGAACTGGATGACT	HindIII	Disruption of the *gsf* gene cluster
AMIS_7260-UF1	GCCAAGCTTCTACCCGATCAACGTCGCAT	HindIII	Disruption of *AMIS_7260*
AMIS_7260-UR1	GCTCTAGACGGCTCCTCGTCGTAATAGA	XbaI	Disruption of *AMIS_7260*
AMIS_7260-DF1	GCTCTAGAAACGATGTGGTCCCGGAGAA	XbaI	Disruption of *AMIS_7260*
AMIS_7260-DR1	GGAATTCAGACGCTCCTGCAGAACTACA	EcoRI	Disruption of *AMIS_7260*
AMIS_7270-UF1	GCCAAGCTTCTGCTTCACTCGTTACCACA	HindIII	Disruption of *AMIS_7270*
AMIS_7270-UR1	GCTCTAGATTCAGCGGTGACCGCACTAA	XbaI	Disruption of *AMIS_7270*
AMIS_7270-DF1	GCTCTAGACTGATCACCGATTCCGGTCT	XbaI	Disruption of *AMIS_7270*
AMIS_7270-DR1	GGAATTCAGAAGTTCTTCGACGTA	EcoRI	Disruption of *AMIS_7270*
AMIS_7280-UF1	GCCAAGCTTTTTCACGGGCATGACCGTGA	HindIII	Disruption of *wpsA*
AMIS_7280-UR1	GCTCTAGACCAGTTCATCGTGCCTCCGAT	XbaI	Disruption of *wpsA*
AMIS_7290-UF1	GCCAAGCTTGAAGTACGTCCGGAACATCTT	HindIII	Disruption of *wpsB*
AMIS_7290-UR1	GCTCTAGACAGAAATAGCACCTGTGCCA	XbaI	Disruption of *wpsB*
AMIS_7290-DF1	GCTCTAGAATCCGTTACGAGGAGTTCCT	XbaI	Disruption of *wpsB*
AMIS_7290-DR1	GGAATTCCACGAGTTCAGGAACGGTTT	EcoRI	Disruption of *wpsB*
AMIS_7300-UF1	GGAATTCACAGCACGTTCTCCACCTTCA	EcoRI	Disruption of *wpsC*
AMIS_7300-UR1	GCTCTAGAGCGGTCGAAAGCCTCGATAGA	XbaI	Disruption of *wpsC*
AMIS_7300-DF1	GCTCTAGAAAGCAGGAGCTGGTGATCGA	XbaI	Disruption of *wpsC*
AMIS_7300-DR1	GCCAAGCTTTACGCGGTGAACGTCCTCTA	HindIII	Disruption of *wpsC*
AMIS_7310-UF1	GCCAAGCTTGTGGTCGTGAACAACCTGCT	HindIII	Disruption of *wpsD*
AMIS_7310-UR1	GCTCTAGAGTCGAAGAACTCGTCGAGGT	XbaI	Disruption of *wpsD*
AMIS_7310-DF1	GCTCTAGACTGTATCGCTACGGCGAGGA	XbaI	Disruption of *wpsD*
AMIS_7310-DR1	GGAATTCAGGAACTCCTCGTAACGGAT	EcoRI	Disruption of *wpsD*
AMIS_7320-UF1	GGAATTCCGCAGCGTATTGCAACTGCT	EcoRI	Disruption of *wpsE*
AMIS_7320-UR1	GCTCTAGACAGCAGCAACAGCAGCACCA	XbaI	Disruption of *wpsE*
AMIS_7320-DF1	GCTCTAGATCCACACCATGGTGAAAGA	XbaI	Disruption of *wpsE*
AMIS_7320-UR1	GCCAAGCTTCATCGACGTTGTCCCAGCCGT	HindIII	Disruption of *wpsE*
AMIS_7330-UF1	GCCAAGCTTTTCGAGTCGGAGCGTGACTT	HindIII	Disruption of *wpsF*

AMIS_7330-UR1	GCTCTAGAGACGGTCTCGTACTTCTCGA	XbaI	Disruption of *wpsF*
AMIS_7330-DF1	GCTCTAGATACGCGGTGAACGTCCTCTA	XbaI	Disruption of *wpsF*
AMIS_7330-DR1	GGAATTCAGCAGGAGCTGGTGATCGA	EcoRI	Disruption of *wpsF*
AMIS_7340-UF1	GCCAAGCTTCCTGCAGGTTCTCATCCAGT	HindIII	Disruption of *wpsG*
AMIS_7340-UR1	GCTCTAGAGACGATCCAGGTGACCAGCA	XbaI	Disruption of *wpsG*
AMIS_7340-DF1	GCTCTAGAGCCCGACGATCTCAATTGGA	XbaI	Disruption of *wpsG*
AMIS_7340-DR1	GGAATTCCCACGACCATCAAATCGTGA	EcoRI	Disruption of *wpsG*
AMIS_7350-UF1	GCCAAGCTTACTCGACAACGGCAAGTCCAA	HindIII	Disruption of *wpsH*
AMIS_7350-UR1	GCTCTAGACAGGTTACGTACGTCCATGGT	XbaI	Disruption of *wpsH*
AMIS_7350-DF1	GCTCTAGATACTACCAGTCGCACGACGA	XbaI	Disruption of *wpsH*
AMIS_7350-DR1	GGAATTCCTCGTTGACGTTGAGAAGCA	EcoRI	Disruption of *wpsH*
AMIS_7360-UF1	GCCAAGCTTGGCAGTGATGTGATCCTGAT	HindIII	Disruption of *wpsI*
AMIS_7360-UR1	AAAACCTGCAGGGAGAACCTCGTTCAGGTACT	Sse8387I	Disruption of *wpsI*
AMIS_7360-DF1	AAAACCTGCAGGCGTAGAGGAGCTCTAGACAC	Sse8387I	Disruption of *wpsI*
AMIS_7360-DR1	GGAATTCACTGGATCAGCACCACGAA	EcoRI	Disruption of *wpsI*
AMIS_7370-UF1	GCCAAGCTTACCATCGACACCACCACGAT	HindIII	Disruption of *wpsJ*
AMIS_7370-UR1	GCTCTAGACATCAGCGGAAAGATCAGCA	XbaI	Disruption of *wpsJ*
AMIS_7370-DF1	GCTCTAGAGCCTTCATGGTTCTGGCGTA	XbaI	Disruption of *wpsJ*
AMIS_7370-DR1	GGAATTCAGATCTCGTCGTAGACCGGTT	EcoRI	Disruption of *wpsJ*
AMIS_7380-DF1	GCTCTAGAGCCCAGGTATTGCGAGACAT	XbaI	Disruption of *wpsK*
AMIS_7380-DR1	GCCAAGCTTCCGGTTCTGTCTCACAAACA	HindIII	Disruption of *wpsK*
AMIS_7390-UF1	GCCAAGCTTAGTCGTTCTCCGGCATGTAT	HindIII	Disruption of *AMIS_7390*
AMIS_7390-UR1	GCTCTAGACAGTACCGATCCATTCACCT	XbaI	Disruption of *AMIS_7390*
AMIS_7390-DF1	GCTCTAGAGAACTGACCATCGACACCAC	XbaI	Disruption of *AMIS_7390*
AMIS_7390-DR1	GGAATTCCATCGAGAGCTTGTAGACGA	EcoRI	Disruption of *AMIS_7390*

[a] The recognition sequences for restriction enzymes are underlined.
